# Large-scale genomic correlations in *Arabidopsis thaliana *relate to chromosomal structure

**DOI:** 10.1186/1471-2164-6-82

**Published:** 2005-06-02

**Authors:** Wayne S Kendal, Brian P Suomela

**Affiliations:** 1 Division of Radiation Oncology, The Ottawa Hospital Regional Cancer Centre, Ottawa, Ontario, K1H 1C4 Canada; 2 The Ottawa Hospital Research Institute, Ottawa, Ontario, K1H 8L6 Canada; 3 Ontario Genomics Innovation Centre, Ottawa, Ontario, K1H 8L6 Canada

## Abstract

**Background:**

The chromosomes of the plant *Arabidopsis thaliana *contain various genomic elements, distributed with appreciable spatial heterogeneity. Clustering of and/or correlations between these elements presumably should reflect underlying functional or structural factors. We studied the positional density fluctuations and correlations between genes, indels, single nucleotide polymorphisms (SNPs), retrotransposons, 180 bp tandem repeats, and conserved centromeric sequences (CCSs) in *Arabidopsis *in order to elucidate any patterns and possible responsible factors for their genomic distributions.

**Results:**

The spatial distributions of all these elements obeyed a common pattern: the density profiles of each element within chromosomes exhibited low-frequency fluctuations indicative of regional clustering, and the individual density profiles tended to correlate with each other at large measurement scales. This pattern could be attributed to the influence of major chromosomal structures, such as centromeres. At smaller scales the correlations tended to weaken – evidence that localized *cis*-interactions between the different elements had a comparatively minor, if any, influence on their placement.

**Conclusion:**

The conventional notion that retrotransposon insertion sites are strongly influenced by *cis*-interactions was not supported by these observations. Moreover, we would propose that large-scale chromosomal structure has a dominant influence on the intrachromosomal distributions of genomic elements, and provides for an additional shared hierarchy of genomic organization within *Arabidopsis*.

## Background

With recent advances in molecular biology we have begun to appreciate both the diversity of genomic elements contained within chromosomes, and the complexity of their distribution. For example, within plant chromosomes there is often a centromeric region of tandemly repeated satellite sequences that is flanked by transposons, retroelements, middle-repetitive elements, and pseudogenes. Beyond this, the genes tends to become more concentrated[1,2]. Other genomic elements like SNPs tend to form smaller scale-invariant clusters within chromosomes, a finding presumably attributable to the random and independent assortment of haplotype blocks, each with distinctive genealogical histories[3,4]. Genes exhibit a clustering similar to SNPs, although the mechanisms at play here are less well understood[5]. Housekeeping genes cluster differently, within regions of high GC content[6,7], and there is evidence that other co-expressed genes cluster within specific chromosomal domains[8].

There are other examples of such non-random positional associations within chromosomes. For example, indels have been reported to collocate with SNPs[9], and transposable elements with regions of low gene density[10]. Eukaryotic genomes appear to be both structurally and functionally organized at multiple hierarchical levels, and these non-random patterns presumably are manifestations of this organization[11].

We performed a detailed analysis of the intrachromosomal distributions of 6 different genomic elements within the *Arabidopsis *genome. In addition to genes, indels, SNPs, and retrotransposons, we studied a number of plant-specific elements catalogued through The *Arabidopsis *Information Resource (TAIR)[12], The Institute for Genomic Research (TIGR)[13] and the *Arabidopsis *genomic repeat database, AtRepBase. This included a class of 180 bp tandem repeats found in the *Arabidopsis *genome[1] and provided by AtRepBase, as well as a class of CCSs as identified by The *Arabidopsis *Genome Initiative [14-19] and catalogued within the TIGR plant repeat database.

## Results

**Figure 1:**
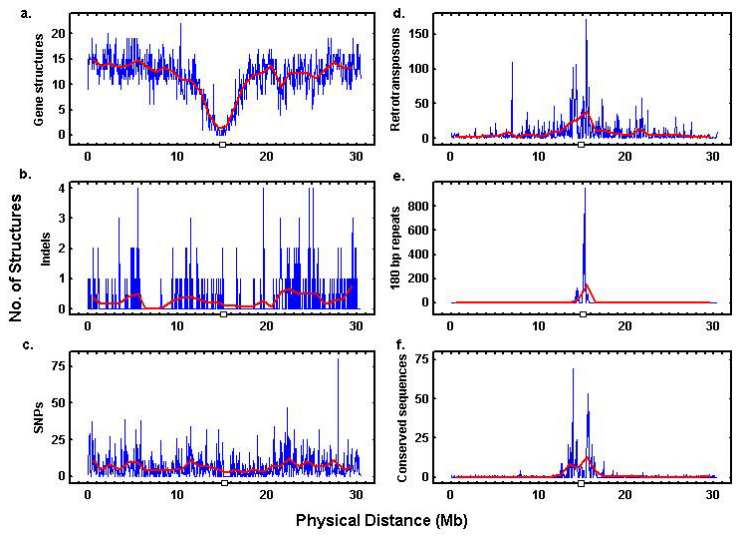
**Density profiles for genomic elements along the length of *Arabidopsis *chromosome 1. **Local densities were estimated based upon 50 kb (blue lines) and 1 Mb (red lines) enumerative bins and the density profiles were plotted as the number of structures per 50 kb length of chromosome *vs*. physical distance as measured from the p-terminal of the chromosome. (a) Genes. (b) Indels. (c) SNPs. (d) Retrotransposons. (e) 180 bp repeats. (f) CCSs. The centromeric region was located at about 15 Mb from the p-terminus. The discontinuity indicated along each X-axis is indicative of the gap in the physical map at this region. Although the lines and axes are drawn continuously in these graphs we must remember that there remains large gaps within the centromeric regions of each chromosome which have not been completely defined.

Figure 1 provides the density profiles of these elements within chromosome 1, as enumerated from both 50 and 1000 kb bins. The smaller bins revealed numerous high-frequency fluctuations; the larger bins more smoothed density fluctuations. In several of the smoothed profiles there were comparatively large density fluctuations coincident with their known positional associations with centromeres[1]. The profiles corresponding to indels and SNPs, however, did not reveal such major centromeric associations. Parenthetically, we should mention that there remain relatively large gaps in the Arabidopsis centromeric regions that have not been completely defined.

We performed spectral analyses of these profiles. Briefly, spectral analysis is a computational method used to study data that fluctuates about a mean value over either time or position. It is based on the mathematical theory of Fourier series, and allows one to resolve density fluctuations like those we observed here into their component harmonics. For each set of density fluctuations we were able to deduce a spectrum, where the intensity (or power) of each harmonic could be plotted *vs*. its particular frequency. Such power spectra can often be exploited to characterize the underlying mechanisms of the fluctuations.

**Figure 2:**
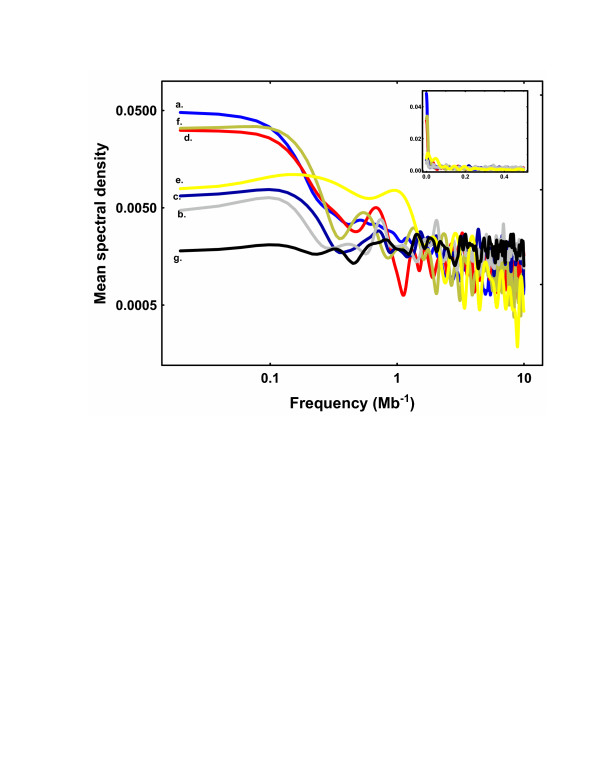
**Power spectra for the fluctuations in positional density from genomic elements in *Arabidopsis *chromosomes. **Mean spectral densities for the fluctuations of each element within individual chromosomes were calculated, normalized, averaged over all 5 *Arabidopsis *chromosomes, and then plotted *vs*. frequency. The power spectra for all 6 genomic elements were qualitatively similar on this log-log plot: the most intense fluctuations were located at the low-frequency ends of the spectra. (a) Genes. (b) Indels. (c) SNPs. (d) Retrotransposons. (e) 180 bp repeats. (f) CCSs. (g) Simulated data from a Poisson distribution. (Insert) Mean Spectral Densities plotted with linear scales to emphasize the concentration of density fluctuations at low frequency.

Figure 2 gives the power spectrum for each genomic element, averaged over all 5 chromosomes. Remarkably, each different spectrum shared a qualitatively similar pattern. The greatest intensity of fluctuation was at the low-frequency end of each spectrum. This low-frequency activity corresponded to the large-scale fluctuations apparent to the smoothed density profiles (Fig. 1). (It would be helpful remind the reader here that frequency in these spectra was inversely related to the scale of the measurement bin size.) Further inspection of these density profiles revealed that the centromeric region had provided a major contribution to these low-frequency density fluctuations for genes, retrotransposons, 180 bp repeats and CCSs.

The low-frequency activity associated with indels and SNPs was admittedly less intense than that observed with the other elements. Yet the highest peaks from each of these power spectra were both qualitatively similar to those demonstrated with the other genomic elements, and distinguishable from simulated random background noise (*P *< 0.006). On the basis of the qualitative similarity between the 6 different power spectra we concluded that the major underlying process(es) that governed the density distributions of the different genomic elements had a similar stochastic basis.

These low-frequency density fluctuations, as observed from 6 genomic elements, indicated a non-random clustering of the individual elements over comparatively large chromosomal regions. Since the indels and SNPs had not exhibited such major concentration changes in the centromeric regions, and since genes, indels, SNPs, and retrotransposons had exhibited low-frequency density fluctuations in non-centromeric regions (Fig. 1), we concluded that non-centromeric chromosomal features may have also contributed to the low frequency activity.

Next we sought to determine whether local concentrations of the different elements might correlate with each other. We calculated the Pearson correlation coefficient *r *between the paired density profiles of the different elements. Given 6 elements, this yielded 15 different permutations. Table 1 provides *r *for all these different permutations, as assessed at 1 Mb intervals. Thirteen of these correlations were statistically significant. Six exceeded |*r*|>0.6 and could be thus considered relatively strong.

**Table 1:** Correlations between different genomic elements in *Arabidopsis*.

Genomic elements compared	*r*^a^	*P *value^b^
retrotransposons *vs*. CCSs	0.87	< 0.00001
retrotransposons *vs*. 180 bp repeats	0.75	< 0.00001
indels *vs*. SNPs	0.68	< 0.00001
180 bp repeats *vs*. CCSs	0.66	< 0.00001
genes *vs*. retrotransposons	-0.65	< 0.00001
genes *vs*. CCSs	-0.63	< 0.00001
SNPs *vs*. retrotransposons	-0.46	0.00005
SNPs *vs*. CCSs	-0.42	0.0004
genes *vs*. 180 bp repeats	-0.41	0.001
indels *vs*. CCSs	-0.40	0.001
SNPs *vs*. 180 bp repeats	0.40	0.002
indels *vs*. retrotransposons	-0.32	0.03
indels *vs*. 180 bp repeats	-0.31	0.05
genes *vs*. SNPs	0.30	NS^c^
genes *vs*. indels	0.23	NS

^a^the correlation coefficient *r *given here represents the quadratic mean taken from all five *Arabidopsis *chromosomes using 1 Mb enumerative bins; ^b^*P *value, probability value; ^c^NS, not significant;

Statistically significant and strong correlations between the positions of indels and SNPs were apparent from our analysis, in agreement with observations from other systems[9]. We also observed a statistically significant and strong negative correlation between genes and retrotransposons, a finding similar to that reported between genes and transposable elements[10].

**Figure 3:**
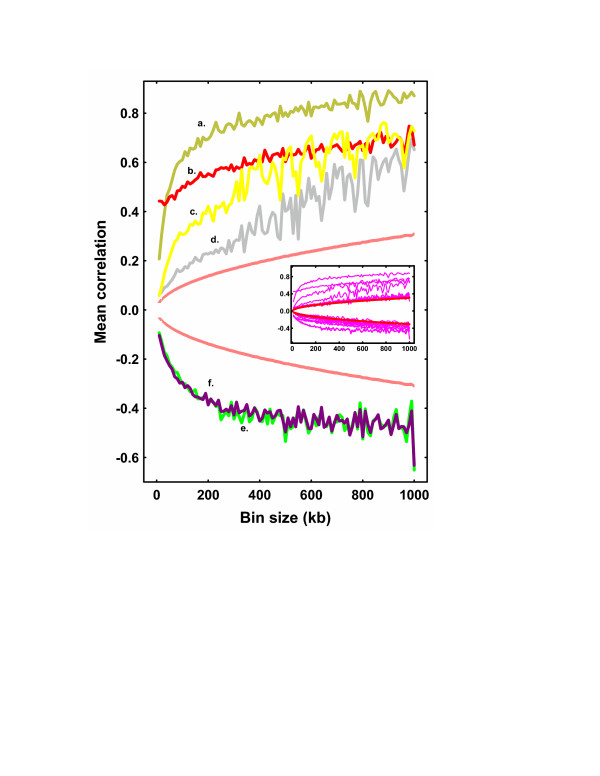
**Mean correlation coefficient *vs*. measurement bin size. **The quadratic mean of *r*, from all 5 *Arabidopsis *chromosomes, was plotted *vs*. a range of measurement bin sizes. The correlations provided here represent the 10 of the stronger relationships: (a) Retrotransposons *vs*. CCSs. (b) Indels *vs*. SNPs. (c) Retrotransposons *vs*. 180 bp repeats. (d) 180 bp repeats *vs*. CCSs. (e) Genes *vs*. retrotransposons (f) Genes *vs*. CCSs. The broken red lines represents the critical values corresponding to *P *= 0.05 and obtained by simulation. (Insert) Mean correlation *vs*. measurement bin size for the envelope of all 15 comparisons. Here the solid red lines represent the critical values corresponding to *P *= 0.05.

We then sought to determine how changes in measurement scale might affect *r*. In Fig. 3, several of the strongest correlations are plotted over a range of measurement scales. Each of these relationships was statistically significant for the full range of scale. Five of these correlation profiles, however, showed marked weakening (*r *≤ 0.2) at bin sizes below 200 kb. The remaining correlation profile, between indels and SNPs (Fig. 3b), however did not reveal as pronounced a decrease at the lower bin sizes. This latter case was an exception to a general trend – the envelope from all 15 correlation pairs (Fig. 3, insert) revealed that the majority of correlations weakened considerably, yet remained statistically significant, at the smaller scales. Because these correlations tended to be strongest at large scales, the associations between elements implied by these correlations were presumably relatively long-ranged. Localized *cis*-interactions thus did not appear to have a major influence on the intrachromosomal distribution of these elements.

## Discussion

To summarize, the density profiles of the 6 diverse genomic elements examined here revealed numerous high frequency and localized fluctuations upon which was superimposed a low frequency and large scale pattern of fluctuation. For 4 of these elements these low frequency fluctuations were concentrated at the centromeric regions of the chromosomes. Spectral analysis confirmed the presence of a dominant low frequency, large scale, component to these density fluctuations in each of the 6 elements, and these fluctuations tended to correlate with each other at large genomic scales. It was apparent on comparative analysis of these three sets of figures that the large scale density fluctuations seen within the density profiles of Fig. 1 were related to the dominant low frequency components evident from the power spectra of Fig. 2, and to the relatively strong large scale correlations seen within Fig. 3. It was also reasonably obvious from Fig 1 that a major (but not exclusive) component to these large scale density fluctuations was derived from centromeric structures.

These large scale correlations were demonstrated with several different genomic elements, which were themselves identified and mapped through an assortment of different methods. The possibility that some form of selection bias might have influenced the results should be considered. It would, however, be difficult to postulate a spurious source of bias to explain such fluctuations and correlations for which the predominant component was evident not only at low frequency and large scales but also with each of the different elements examined. Such a putative bias would necessarily have to be associated with the centromeres. Despite the large undefined gaps in centromeric regions that remain, sufficient portions of the centromeres have been defined so as to provide us with strong evidence for the associated centromeric density changes for gene structures, retrotransposons, 180 bp repeats and conserved centromeric sequences[1,2]. We would propose instead that the most plausible explanation for these low frequency density fluctuations, and large scale correlations, would be itself the large scale structural features of *Arabidopsis *chromosomes.

The persistent correlation between indels and SNPs at smaller measurement scales (Fig. 3c) warrants further consideration. These two elements have been shown to correlate with each other within the HLA region of the human genome, a observation that might be attributed to repetitive insertions and deletions, imperfect segmental duplications or adjacent nucleotide changes[20] – all of these presumably localized processes. Our observation of a modest 20% increase in the respective value of *r *with increased bin size indicated that in *Arabidopsis *a component of this correlation could be attributed to large-scale chromosomal features. At the same time, we could not exclude the possibility that the persistence of this correlation at small scales might not be attributable to localized interactions, but rather to methodological artifact since the positional cloning used to detect both SNPs and indels for the TAIR database might have allowed selection bias.

We also observed that the densities of genes and retrotransposons exhibited statistically significant and strong negative correlation, a finding consistent with previous observations with transposable elements. This has been explained by a presumed selection against the disruption of gene expression by transposable elements[10]. As with the other associations discussed here, it was difficult to reconcile explanations based upon localized processes with the decreased strength of the correlation at small measurement scales. The hypothesis that large-scale chromosomal structure could influence the spatial distribution of a variety of chromosomal elements seemed to provide a more plausible explanation.

## Conclusion

We have demonstrated that the physical distributions of genomic elements within *Arabidopsis *chromosomes were highly heterogeneous, yet shared a common distributional pattern. The density profiles of each element exhibited low-frequency fluctuations indicative of regional clustering, and these density profiles tended to correlate with each other at large measurement scales. This was the dominant pattern underlying the positional distributions of all 6 genomic elements examined. Localized *cis*-interactions between different elements had a comparatively minor, if any, influence on the intrachromosomal distribution of genomic elements. This study demonstrated an additional hierarchy of eukaryotic genomic organization that was both common to a diverse set of genomic elements, and associated with major chromosomal features.

## Methods

### Data abstraction

The *Arabidopsis thaliana *data used in this study was obtained from a variety of sources: The positions of genes were obtained from the National Center for Biotechnology Information (NCBI) website  and localization of indels and SNPs was provided by TAIR . TIGR provided sequence information for *Arabidopsis *pseudochromosomes ) as well as for retrotransposons and CCSs . Sequences for the 180 bp repeats were obtained from AtRepBase . Localization of retrotransposons, CCSs and the 180 bp repeats was performed by running BLASTN as provided at the TIGR website. In order to be as inclusive as possible, the stringency of these matches was kept low; as well, filters were used to exclude redundant matches. We thus accessed the positions of 29,826 genes, 1,732 indels, 20,008 SNPs, 20,564 retrotransposons, 14,064 tandem repeats, and 3,896 CCSs.

Since TAIR and TIGR act as repositories for the publicly available *Arabidopsis *data, a number of different methods had been used by many different investigators to identify the positions of the various genomic elements: SNPs and indels were identified by positional cloning of the available bacterial artificial chromosomes (BACs)[21], and by the large-scale analysis of expressed sequence tags from different accessions of *Arabidopsis*[22].

In our analysis the 5 *Arabidopsis *chromosomes were divided into non-overlapping, equal-sized, sequential bins and the numbers of *p*-termini for the structure of interest were enumerated for each bin. A range of bin sizes was employed, from 10 to 1,000 kb. Density profiles for the individual structures thus obtained were subjected to the additional analyses detailed below.

### Spectral analysis

Density profiles for each genomic feature were parsed into 50 kb bins and padded with zeros to a length of 1024 data points. A 15% split-cosine-bell taper was employed on the data at the beginning and end of each sequence, the mean was subtracted, the data de-trended, and a 15-point Hamming data window was employed for data smoothing. The spectral density of each structure was calculated using a Cooley-Tukey fast Fourier transform and then normalized. The spectral densities were averaged over all 5 *Arabidopsis *chromosomes and then plotted *vs*. frequency. As a control for these analyses we performed Monte Carlo simulations for a random (Poisson) distributed genomic element within five chromosomes with a density comparable to that observed with genes. These data were then processed as above to yield a spectral density. A more extensive set of simulations was done to estimate the critical values for the amplitude of individual peaks expected from a random distribution of elements, and with parameters to emulate both the chromosomal densities of elements and the lengths of the individual chromosomes.

### Correlation analysis

The Pearson correlation coefficient *r *was calculated between different density profiles over a range of bin sizes (10 to 1000 kb), and then plotted *vs*. bin size. We also performed scatter plots between the density profiles to exclude pronounced nonlinear relationships between variables (data not provided). Because *r*^2 ^is additive, whereas *r *is not, the quadratic mean was used to average correlations from the 5 *Arabidopsis *chromosomes. Critical values for the quadratic means were estimated by Monte Carlo simulations, based upon the premise of a *t*-distribution for the quantity, , where *n *represents the number of data points[23].

## List of abbreviations used

SNPs – single nucleotide polymorphisms; CCSs – conserved centromeric sequences; NCBI – National Center for Biotechnology Information; TAIR – The *Arabidopsis *Information Resource; TIGR – The Institute for Genomic Research; AtRepBase – *Arabidopsis *genomic repeat database; BACs – bacterial artificial chromosomes; *P *value – probability value; NS – not significant; *vs*. – versus; bp – base pairs; kb – kilobases; HLA – human leukocyte associated antigens; GC – guanidine cytosine; BLASTN – basic local alignment search tool for nucleotides ; PCR – polymerase chain reaction; *r *– Pearson correlation coefficient.

## Authors' contributions

WSK conceived this study, conducted the spectral and correlation analyses, and drafted the manuscript. BPS participated in the design of the study, and conducted the bioinformatic data abstraction. Both authors read and approved the final manuscript.
